# Excess all-cause and influenza-attributable mortality in Europe, December 2016 to February 2017

**DOI:** 10.2807/1560-7917.ES.2017.22.14.30506

**Published:** 2017-04-06

**Authors:** Lasse S Vestergaard, Jens Nielsen, Tyra G Krause, Laura Espenhain, Katrien Tersago, Natalia Bustos Sierra, Gleb Denissov, Kaire Innos, Mikko J Virtanen, Anne Fouillet, Theodore Lytras, Anna Paldy, Janos Bobvos, Lisa Domegan, Joan O’Donnell, Matteo Scortichini, Annamaria de Martino, Kathleen England, Neville Calleja, Liselotte van Asten, Anne C Teirlinck, Ragnhild Tønnessen, Richard A White, Susana P Silva, Ana P Rodrigues, Amparo Larrauri, Inmaculada Leon, Ahmed Farah, Christoph Junker, Mary Sinnathamby, Richard G Pebody, Arlene Reynolds, Jennifer Bishop, Diane Gross, Cornelia Adlhoch, Pasi Penttinen, Kåre Mølbak

**Affiliations:** 1 Statens Serum Institut, Copenhagen, Denmark; 2 Scientific Institute of Public Health, Brussels, Belgium; 3 National Institute for Health Development, Tallinn, Estonia; 4 National Institute for Health and Welfare, Helsinki, Finland; 5 French Public Health Agency (Santé Publique France), Saint-Maurice, France; 6 Hellenic Centre for Disease Control and Prevention, Athens, Greece; 7 National Public Health Center, Budapest, Hungary; 8 Health Service Executive - Health Protection Surveillance Centre, Dublin, Ireland; 9 Dipartimento Epidemiologia del S.S.R., Lazio – ASL Roma 1, Rome, Italy; 10 Ministero della Salute, Rome, Italy; 11 Ministry of Health, Pieta, Malta; 12 National Institute of Public Health and the Environment (RIVM), The Netherlands; 13 Norwegian Institute of Public Health, Oslo, Norway; 14 Instituto Nacional de Saúde Doutor Ricardo Jorge, Lisbon, Portugal; 15 CIBER Epidemiología y Salud Pública (CIBERESP) Instituto de Salud Carlos III, Madrid, Spain; 16 The Public Health Agency of Sweden, Stockholm, Sweden; 17 Federal Statistical Office, Neuchâtel, Switzerland; 18 Public Health England, Colindale, United Kingdom; 19 Health Protection Scotland, Glasgow, United Kingdom; 20 WHO Regional Office for Europe, Copenhagen, Denmark; 21 European Centre for Disease Prevention and Control (ECDC), Stockholm, Sweden

**Keywords:** Mortality, influenza, influenza virus A(H3N2), surveillance, Europe, EuroMOMO

## Abstract

Since December 2016, excess all-cause mortality was observed in many European countries, especially among people aged ≥ 65 years. We estimated all-cause and influenza-attributable mortality in 19 European countries/regions. Excess mortality was primarily explained by circulation of influenza virus A(H3N2). Cold weather snaps contributed in some countries. The pattern was similar to the last major influenza A(H3N2) season in 2014/15 in Europe, although starting earlier in line with the early influenza season start.

During winter seasons in Europe, an increase in all-cause mortality is often observed. This excess mortality may vary considerably between countries, by age group and from one season to another [1-5]. Circulation of influenza virus, in particular with the subtype A(H3N2), has been shown to be the main seasonal driver of excess mortality, particularly among the elderly (≥ 65 years of age), but other factors such as other respiratory agents and extreme cold weather may contribute as well [6-10]. In the current 2016/17 winter season, from the end of 2016 and until calendar week 8/2017, marked excess all-cause mortality was observed in many countries participating in the network for European monitoring of excess mortality for public health action (EuroMOMO), particularly in people 65 years and older, but also among those aged 15–64 years. Here we describe the excess all-cause mortality and estimate the influenza-attributable mortality for the current winter season until calendar week 8/2017 in Europe.

## European monitoring of excess mortality for public health action

Since 2009, the EuroMOMO network (www.euromomo.eu) has monitored weekly all-cause age group-specific excess mortality in several European countries. EuroMOMO uses a statistical algorithm, which allows for comparison and pooling of national and regional mortality data [4]. More recently, influenza activity (IA) data, based on reported national rates of influenza-like illness (ILI) or acute respiratory infection (ARI), or, if not available, based on reported intensity of IA (categorised as low, medium, high, very high), is used to estimate the burden of influenza-attributable mortality, applying a statistical algorithm known as FluMOMO [11].

### Estimation of all-cause mortality

Countries in the EuroMOMO network collected weekly data on the number of deaths from all causes, and excess (deviation from baseline) all-cause number of deaths was estimated using the EuroMOMO statistical algorithm described previously [4]. Staff at the EuroMOMO hub at Statens Serum Institut in Copenhagen, Denmark, compiled weekly data from individual countries and conducted a pooled analysis using an age-stratified method [7], which included data from 19 European countries or regions (Belgium, Denmark, England (United Kingdom (UK)), Estonia, Finland, France, Greece, Hungary, Ireland, Italy, Malta, the Netherlands, Norway, Portugal, Scotland (UK), Spain, Sweden, Switzerland and Wales (UK)). We used z-scores to standardise outputs enabling comparisons of mortality patterns between different countries and between different time-periods. Estimates are shown as totals (all age groups) and stratified by age groups (< 5, 5–14, 15–64 and ≥ 65 years). The pooled analysis covers all-cause mortality up to and including calendar week 8/2017, based on data received by week 9/2017.

We also calculated the cumulative excess all-cause mortality for the current winter season and compared it with the previous winter seasons of 2013/14, 2014/15 and 2015/16. Winter seasons are defined as the period between calendar week 40 in a given year and week 20 in the following year.

### Estimation of influenza-attributable mortality

The number of influenza-attributable deaths in the EuroMOMO network countries was estimated using the FluMOMO algorithm, based on weekly IA data (ILI, ARI or intensity data, as available) from the participating 19 EuroMOMO countries, retrieved from the TESSy database at the European Centre for Disease Prevention and Control (ECDC) [12]. The model is a multiplicative Poisson regression time-series model with over-dispersion and International Organization for Standardization (ISO)-week as time unit. As in the EuroMOMO model, the multiplicative residual variance is post-regression corrected for skewness by applying a 2/3-power correction [13]. As the dominant type/subtype of influenza viruses circulating varies from season to season, a separate effect of IA for each season is used. To adjust for a possible confounding effect of temperature, an explanatory variable reflecting ambient temperature deviation from expected normal temperature is included in the model, obtained for each of the countries from the respective National Oceanic and Atmospheric Administration (NOAA). Further, two weeks delayed effects of the explanatory variables are also included in the model. The model estimates both a baseline and the effect of IA and temperature simultaneously, i.e. controlled for one another. IA data from the same countries and for the same time period as used to calculate the all-cause mortality, mentioned above, was used.

Based on the estimated number of deaths, mortality rates were calculated using national population data downloaded from EuroStat, as at 1 January 2017, and linearly interpolated.

### Influenza sentinel surveillance data

Weekly proportions of primary care sentinel specimens testing positive for influenza in the participating EuroMOMO network countries that had experienced excess mortality in the 2016/17 winter season were analysed and compared with previous seasons since 2011/12 [14].

## Results

All-cause mortality started to exceed normal levels in Portugal around calendar week 50/2016. Soon after, excess mortality was also detected in many other EuroMOMO network countries, including the following (mentioned in alphabetic order): Belgium, England (UK), Finland, France, Greece, Ireland, Italy, Malta, the Netherlands, Norway, Scotland (UK), Spain, Switzerland and Wales (UK). Countries in southern Europe experienced particularly high excess mortality levels. The observed excess all-cause mortality was most prominent in individuals aged 65 years and older, but some countries also observed excess deaths among those aged 15–64 years. At week 8/2017 mortality levels were still elevated in most of the reporting countries and only three countries, Denmark, Estonia and Hungary had not observed any significant excess mortality in 2016/17.

**Figure 1:**
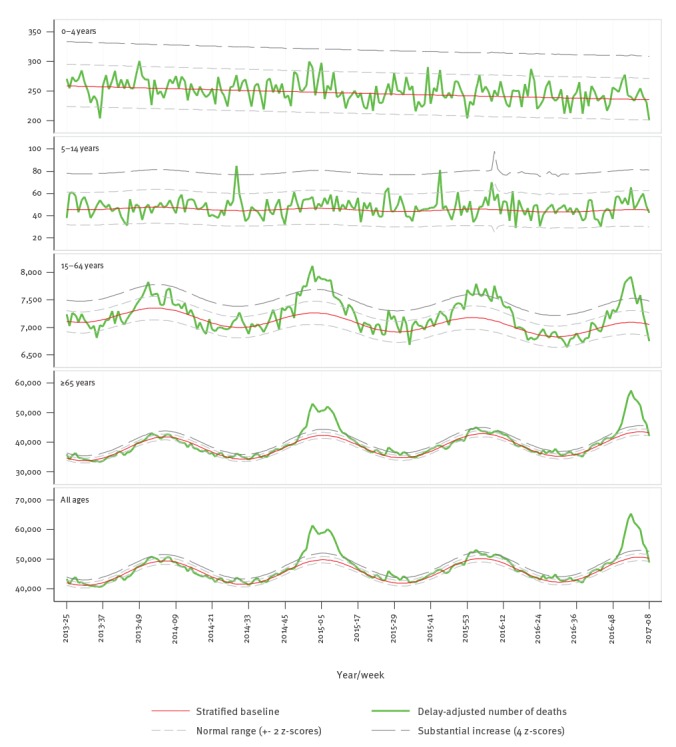
Number of all-cause deaths by week and modelled baseline from pooled analysis of data, participating EuroMOMO countries/regions, calendar week 25/2013 until week 8/2017

EuroMOMO: European monitoring of excess mortality for public health action; UK: United Kingdom.

Evaluation of the pooled excess all-cause mortality of the 19 participating European countries/regions revealed a sharp rise in mortality among individuals aged 65 years and older, starting around the turn of the year and exceeding 4 z-scores above baseline in calendar week 2/2017 (Figure 1).

Participating countries: Belgium, Denmark, England (UK), Estonia, Finland, France, Greece, Hungary, Ireland, Italy, Malta, the Netherlands, Norway, Portugal, Scotland (UK), Spain, Sweden, Switzerland and Wales (UK).

**Figure 2:**
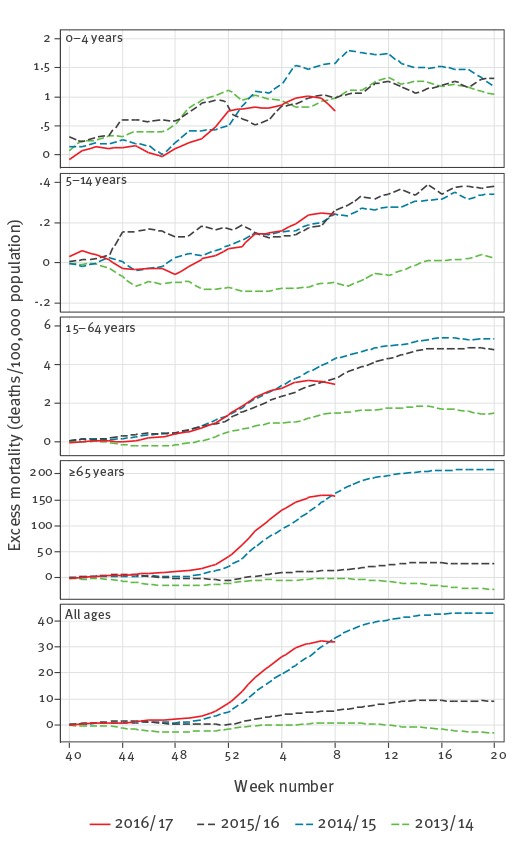
Cumulated pooled excess all-cause mortality, participating EuroMOMO countries/regions, winter seasons 2013/14, 2014/15, 2015/16 and 2016/17 (until week 8/2017)

EuroMOMO: European monitoring of excess mortality for public health action; UK: United Kingdom.

**Figure 3:**
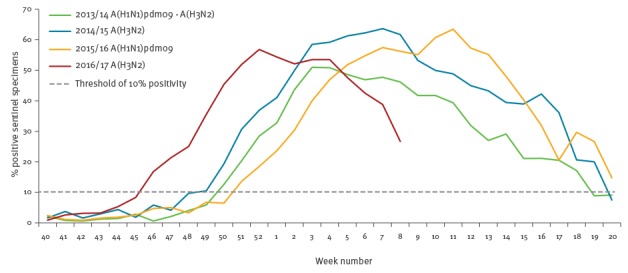
Weekly proportions of influenza-positive primary care sentinel specimens and threshold of 10% positivity, participating EuroMOMO countries/regions^a^, winter seasons 2013/14 to 2016/17 (until week 8/2017)

The cumulated pooled excess (deviation from baseline) all-cause mortality observed in the 2016/17 winter season compared with the previous three winter seasons (Figure 2) showed that excess mortality in those aged 65 years and older reached considerable excess levels. The pattern resembled that of the severe 2014/15 season albeit with a few weeks´ earlier onset of the increase, in line with an earlier onset of influenza virus circulation in 2016/17 (Figure 3).

Participating countries: Belgium, Denmark, England (UK), Estonia, Finland, France, Greece, Hungary, Ireland, Italy, Malta, the Netherlands, Norway, Portugal, Scotland (UK), Spain, Sweden, Switzerland and Wales (UK).

Winter seasons: period between calendar week 40 in a given year and week 20 in the following year.

EuroMOMO: European monitoring of excess mortality for public health action.

^a^ Countries included in the graph, that experienced excess mortality in the 2016/2017 season, are: Belgium, Finland, France, Ireland, Italy, Malta, The Netherlands, Norway, Portugal, Spain, Sweden, Switzerland and United Kingdom.

Week 53/2015 excluded.

Winter seasons: period between calendar week 40 in a given year and week 20 in the following year.

**Figure 4:**
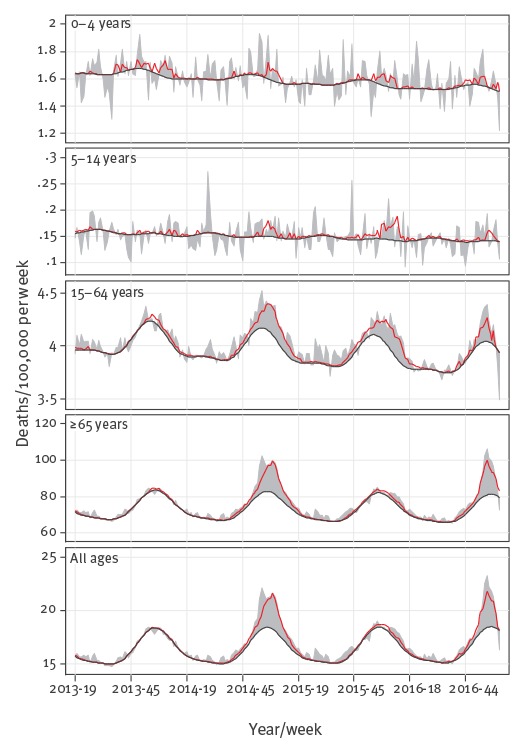
Weekly all-cause and influenza-attributable mortality rates (deaths per 100,000 people per week), participating EuroMOMO countries, winter seasons 2013/14 to 2016/17 (until week 8/2017)

Seasonal variation in excess mortality estimates for the 19 participating countries/regions, derived from the FluMOMO model output, could primarily be attributed to seasonal variation in influenza activity (Figure 4).

EuroMOMO: European monitoring of excess mortality for public health action; UK United Kingdom.

Estimates are calculated with the FluMOMO model (see main text for details).

The shaded grey areas represent deviations in expected all-cause deaths from the estimated baseline. The red curves signify mortality attributable to influenza activity.

Participating countries: Belgium, Denmark, England (UK), Estonia, Finland, France, Greece, Hungary, Ireland, Italy, Malta, the Netherlands, Norway, Portugal, Scotland (UK), Spain, Sweden, Switzerland and Wales (UK).

Winter seasons: period between calendar week 40 in a given year and week 20 in the following year.

In this model, IA seemed to be an important driver of the observed overall excess winter mortality (Table). The estimated pooled excess all-cause winter mortality among people aged 65 years and older according to the EuroMOMO model reached 158 (95% confidence interval (CI): 153–162) deaths per 100,000 population for the 2016/17 season (until week 8/2017), compared with 208 (95% CI: 202–214) for the whole season of 2014/15, and they were well above the 2013/14 and 2015/16 seasons estimates (Table). The same pattern was observed for the estimated cumulated influenza-attributable mortality using FluMOMO, with 137 (range: 76–302) deaths per 100,000 population in the 2016/17 winter season (until week 8/2017), compared with 185 (range: 82–311) in the winter season of 2014/15 (Table).

**Table:** Estimates of cumulated pooled excess (deviation from baseline) all-cause mortality rates^a^ and cumulated combined influenza-attributable mortality^b^ rates, participating EuroMOMO countries, winter seasons 2013/14 to 2016/17 (until week 8/2017)

Individuals ≥ 65 years	Cumulated pooled excess all-cause mortality^a^ (excess deaths per 100,000 people)			Cumulated combined influenza-attributable mortality^b^ (excess deaths per 100,000 people)		
Winter season period	CMR	95% CI		CMR	min max	
Week 40/2013–Week 20/2014	-23	-29	-17	30	0	88
Week 40/2014–Week 20/2015	208	202	214	185	82	311
Week 40/2015–Week 20/2016	28	22	34	45	0	207
Week 40/2016–Week 8/2017	158	153	162	137	76	302

CI: confidence interval; CMR: cumulative mortality rates; EuroMOMO: European monitoring of excess mortality for public health action; UK: United Kingdom.

^a^ Based on EuroMOMO algorithm.

**^b^** Based on FluMOMO algorithm.

Estimates are based on data reported by calendar week 9/2017, and may differ from previously reported season estimates.

Participating countries: Belgium, Denmark, England (UK), Estonia, Finland, France, Greece, Hungary, Ireland, Italy, Malta, the Netherlands, Norway, Portugal, Scotland (UK), Spain, Sweden, Switzerland and Wales (UK).

Winter seasons: period between calendar week 40 in a given year and week 20 in the following year.

## Discussion

As at week 8/2017 of the 2016/17 winter season in Europe, influenza virus A(H3N2) predominated and circulated widely, and a large number of European countries experienced markedly increased mortality levels, particularly in their elderly populations. The EuroMOMO pooled analysis showed that this years’ excess mortality started earlier than what was observed across Europe during the previous influenza A(H3N2) predominant season in 2014/15. The estimates of mortality attributable to IA, from FluMOMO, showed a similar pattern. The pooled estimates of all-cause and influenza-attributable mortality in 2016/17 at week 8/2017 were slightly lower than the estimates from the 2014/15 season, but this may change as the season progresses.

Pooled estimates may mask important local differences in influenza-attributable mortality, including effects of extreme temperatures in some countries. Indeed, many parts of Europe were affected by very cold weather in January 2017 which may have had an impact on the all-cause excess mortality. Therefore, we estimated the influenza-attributable deaths among older adults adjusting for extreme temperatures. We found that throughout Europe the excess mortality was mainly explained by the early peak and widespread circulation of influenza A(H3N2), the influenza virus most frequently associated with fatal influenza in the elderly [14,15]. Indeed, influenza morbidity and mortality put a significant strain on health facilities and hospitals in many countries across Europe in the first weeks of 2017 [14].

The scenario during this influenza season in Europe seemed remarkably similar to the season in 2014/15. That season was also characterised by a sharp rise in mortality in the elderly coinciding with widespread circulation of influenza A(H3N2) virus in many countries, as also detected and reported through the EuroMOMO mortality monitoring system [5]. The A(H3N2) virus strain that circulated in 2014/15 had drifted considerably from the strain chosen as the A(H3N2) component in the seasonal vaccine, possibly also contributing to the excess mortality among the elderly, the key target group for vaccinations in Europe. Interim estimates of the 2016/17 vaccine effectiveness have shown only a moderate effectiveness against influenza A(H3N2) both in Europe [16,17] and in North America [18,19]. Therefore, rapid use of neuraminidase inhibitors and supportive care for any confirmed or probable case of influenza infection should be considered for the management of vaccinated as well as non-vaccinated patients at risk of developing severe illness and complications.

EuroMOMO has proven a valuable network for timely detection and reporting of excess all-cause mortality across many parts of Europe in a coordinated manner. In this report we also provide for the first time results from the FluMOMO statistical model pilot, which enables us to demonstrate how IA affects mortality, adjusted for the confounding effect of deviations from expected ambient temperatures, like extreme cold temperatures. This is an important advance in the rapid risk assessment of seasonal influenza. Our approach and experiences in ‘real-time’ monitoring of excess mortality may contribute to improving regional and global estimation of the severity of ongoing influenza seasons, or a developing influenza pandemic, in a timely manner. Based on its relatively simple technical and operational features, the use of the FluMOMO model may provide a user-friendly, yet powerful, tool for rapid public health action.

Despite the results presented here, further validation of the described approach is warranted. For instance, we need to explore the use of different influenza parameters, as clinical indicators of respiratory disease such as ILI and ARI on their own may not be the best indicators of influenza-attributable mortality and influenza virus circulation. Nonetheless, the use of such routine influenza surveillance data has proven valuable for the monitoring of the community impact of influenza at the European level [20]. The practicalities of retrieving national IA data directly from TESSy at ECDC [12] need further evaluation and optimisation before the procedure can be set up and operated on a routine basis. We will continue to conduct further in-depth analysis and validations of the FluMOMO model, aiming to develop an even more reliable and time-effective tool to monitor the severity of seasonal influenza in Europe and beyond.

The winter season has not ended yet and additional excess mortality may still emerge. We have noted some heterogeneity in mortality patterns across participating countries, which may reflect some real differences between countries, possibly related to varying levels of influenza virus circulation, due to country-specific population susceptibility or other contributing factors, such as differences in influenza vaccine policy and uptake. We will, therefore, continue to monitor the situation closely in the coming weeks and months.
